# Describing the gingival involvement in a sample of 182 Italian predominantly oral mucous membrane pemphigoid patients: A retrospective series

**DOI:** 10.4317/medoral.21431

**Published:** 2017-02-04

**Authors:** Paolo G. Arduino, Roberto Broccoletti, Mario Carbone, Davide Conrotto, Erica Pettigiani, Silvia Giacometti, Alessio Gambino, Alessandra Elia, Marco Carrozzo

**Affiliations:** 1 Department of Surgical Sciences, Oral Medicine Section, CIR-Dental School, University of Turin, Turin, Italy; 2 Department of Oral Medicine, School of Dental Sciences, University of Newcastle upon Tyne, Newcastle upon Tyne, UK

## Abstract

**Background:**

The oral cavity has been frequently described as the only site of involvement or as the first manifestation of mucous membrane pemphigoid (MMP), being the gingival tissues often involved, but usually this has been effusively detailed in limited case series. This is a retrospective evaluation of the gingival involvement in 182 Italian patients with oral MMP.

**Material and Methods:**

The diagnosis of MMP was established by both clinical morphology and direct immunofluorescence finding. Patient information (age, gender, risk factors and medical status) and parameters of manifestation (lesions’ distribution, site and type) were detailed.

**Results:**

The mean age was 62 years for women (n=137) and 67 years for men (n=45). Patients had several sites of oral involvement; the gingiva was the most common one, affecting 151 patients (82.96%; 119 f - 32 m). Female subjects had more possibilities to develop gingival lesions than male patients (*P* = 0.005). Sixty-five patients (35.7%; 58 f - 7 m) had pure gingival involvement. Patients with lower gingival involvement statistically had more complaints (*P* = 0.006).

**Conclusions:**

This report is one of the largest about predominantly oral MMP cases, detailing the very frequent gingival involvement; this could be crucial not only for oral medicine specialists but also for primary dental healthcare personnel and for periodontists.

**Key words:**Mucous membrane pemphigoid, gingival status, clinical features.

## Introduction

Mucous membrane pemphigoid (MMP) describes a mixed group of chronic and inflammatory diseases presenting macroscopically with various oral, ocular, skin, genital, naso-pharyngeal and laryngeal lesions. MMP is a sub-epithelial blistering affection, microscopically characterized by linear deposition of immunoglobulins (mainly IgG or C3) along the epithelial basement membrane (1).

There is an evident degree of variability in the clinical and immunological features of MMP, suggesting the existence of multiple phenotypic variants (2). From a diagnostic and therapeutic view, it might be of significant advantage to discriminate a clinical subset of MMP, termed oral pemphigoid, in which the disease is mainly limited to the oral cavity and patients have a better prognosis compared to other variants (3).

The oral cavity has been repeatedly described as the only site of involvement or as the first manifestation of the disease (4), being the gingival tissues often involved, but usually this has been effusively detailed in limited case series. Gingival MMP is usually described with diffuse erythematous lesions, blisters, erosions or ulcerations, mainly located on the attached gingiva and on the palate; the occurrence of epithelial desquamation, erythema, and erosive lesions on the gingival tissue is described in literature as “desquamative gingivitis” (DG) (5-7). It has been reported that DG could play a role in increasing the long-term risk for periodontal tissue breakdown at specific sites, but there is conversely inadequate evidence to support this (8,9). Recently, we also reported that subjects diagnosed with MMP have higher possibilities of gingival and periodontal inflammation than healthy controls (7).

In this study, we reviewed different gingival characters of 182 Italian patients with predominantly oral MMP. To date, this is the biggest series of patients with oral MMP ever reported from Italy.

## Materials and Methods

From a standardised computerised databank, the files of subjects who had been originally referred to the Oral Medicine Section of University of Turin, Italy, for the diagnosis and management of MMP, from January 1991 to May 2014, were reviewed and significant data designated. At baseline we detailed: histological diagnosis, ethnicity, age and sex, smoking and alcohol use, oral symptoms, gingival clinical features, other sites of oral involvement and extra-oral appearances.

The present analysis was approved by the Institutional Review Board of CIR-Dental School, University of Turin, and conducted according to the Declaration of Helsinki.

- Diagnostic criteria

The diagnosis of MMP has to be established by both clinical morphology and a direct immunofluorescence finding of linear deposition of immunoglobulins, or complement components, at the epithelial basement membrane zone. Each specimen was blindly re-examined by an expert oral pathologist, not involved directly in the study.

- Data analysis

All data collected were assessed using descriptive statistics, and continuous variables expressed as mean ± SD. The statistical evaluation was made by Pearson’s chi-squared test (χ2) and were considered significant for *P* values ​​< 0.05. All analyses were completed using SPSS software (SPSS for windows, version 11, SPSS inc, Chicago, IL, USA).

## Results

A total of 217 patients have been initially selected but 35 were excluded because of a diagnosis not completely conformed. Ultimately, 182 subjects were described, of whom 137 were women (~75%) and 45 men (f : m = 3.04 : 1).

The mean age at presentation was 63.52 years (± 14.15); in particular, it was 66.95 years for men (± 14.19), and 62.14 years for women (± 13.77), showing a high percentage of affected subjects between 5th (48 patients) and 6th decades (55 patients).

Follow-up varied from 6 to 222 months, with a median of 54.50 months; in particular, it was 50 months for men (± 46.97), and 55.96 months for women (± 51.12). Sixty-eight patients (37.4%) were followed-up for less than 2 year (median of 9.5 months), 55 (30.2%) for a long lasting period of 5 years (median of 45 months) and 59 (32.4%) for more than 5 years (median of 108 months).

Most of the subjects did not smoke (95.4%) and many of them did not drink alcohol (87.7%).

The medical files of the selected patients comprised mainly hypertension (25%), cardiac (13.8%) and gastrointestinal (17%) diseases, hypothyroidism (10%), allergies and osteoporosis (both 7%), neoplastic diseases (3.84%), autoimmune diseases (3%), psoriasis (2%). Neoplastic disorders (detailed at baseline) were: breast cancer (n=3), melanoma (1), lung cancer (1), and prostate cancer (2). Medications taken were principally anti-hypertensive (22.1%) and anxiolytics (7.9%).

Direct immunofluorescence (IF) investigations showed characteristic continuous linear deposits of IgG (93.4%) and C3 (82.8%), seldom IgA (12.9%), in the epithelial basement membrane zone in all MMP patients.

The gingiva was the most usual site of involvement, affecting 151 patients (82.96%; 119 f - 32 m), followed by the palate (34%), buccal mucosa (25.3%), alveolar ridge (11%), tongue (7.7%) and labial mucosa (7.7%). Female subjects had more possibilities to develop gingival lesions than male patients (*P* = 0.005). The involvement of the upper gingiva was reported in 140 patients (112 f - 28 m), while the lower was involved in 130 cases (100 f – 30 m). The upper involvement was also more frequently reported in female (*P * = 0.007), while the lower one presented no differences between different genders (*P * = 0.166).

Sixty-five patients (35.7%; 58 f - 7 m) had pure gingival involvement.

**Figure 1:**
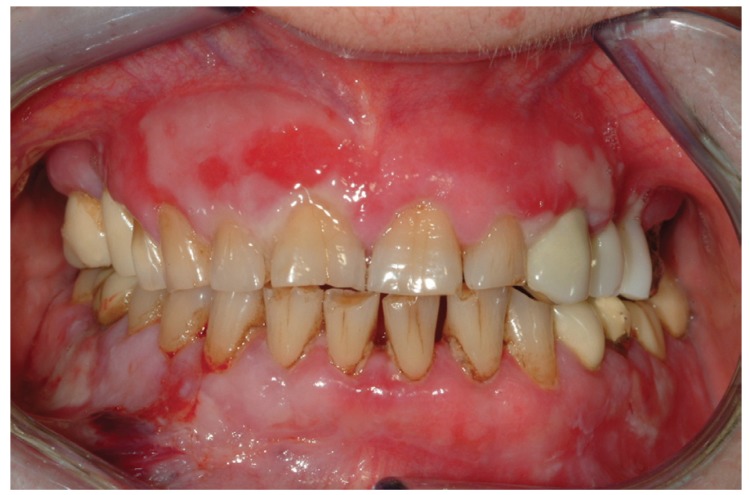
Typical gingival features in a predominantly MMP patient.

The clinical characters of MMP gingival lesions are described in  Table 1 (Fig. 1). Gingival redness is the major clinical appearance (95%); the vestibular site is more often involved, twice than the palatal/lingual one. Blistering and erosion affect usually 60% of cases.

**Table 1:**
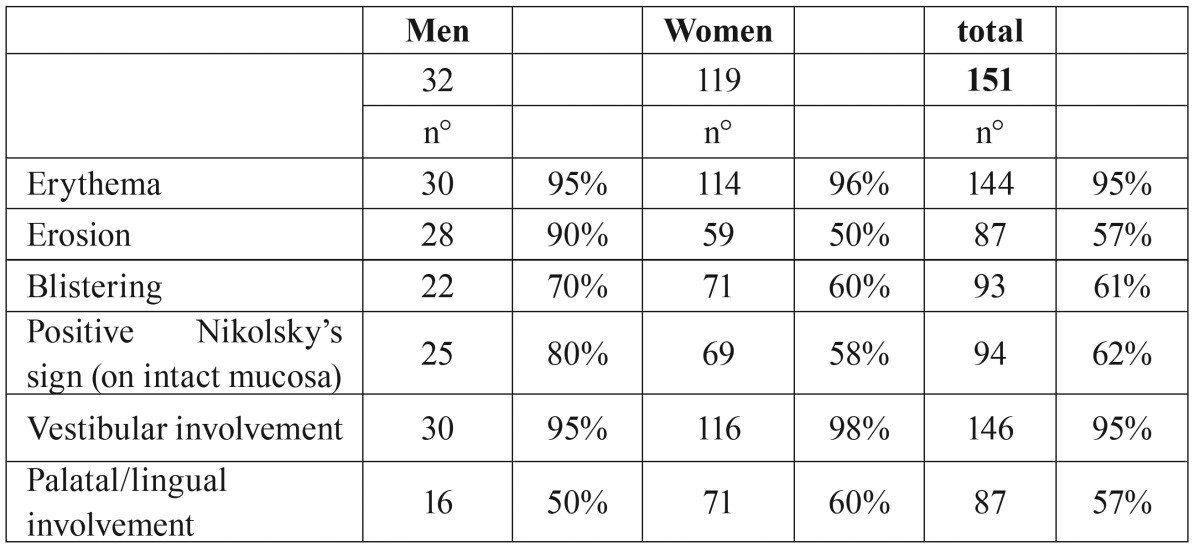
Gingival clinical features of mucous membrane pemphigoid patients (tot patients=182; with gingival involvement=151).

One hundred and eighteen patients (89 f - 29 m) also reported different kind of symptoms, (e.g.: pain, the most common complaint, but furthermore burning, irritation or bleeding), with no statistical difference between male and female (*P * = 0.517). Regarding the site of involvement (oral and extra-oral), only patients with lower gingival involvement statistically had more complaints (*P * = 0.006)

Less than 30% of patients had also extra-oral manifestation; of those, 21 (9 f - 12 m) had ocular involvement, 29 had skin involvement (18 f - 11 m), and 24 had ENT involvement (13 f - 11 m). Male subjects statistically developed more frequently ocular and ENT lesions (respectively *P * = 0.001 and *P * = 0.012).

## Discussion

Since oral lesion of MMP had always been described to occur more commonly at sites exposed to chronic inflammation or trauma, it is not unforeseen that the most reliable and the most distinguishing features of this rare disease are those of DG. The gingiva is generally one of the oral sites with the greatest incidence after the palate and the buccal mucosa (10). In this study, almost 83% of subjects suffered from gingival lesions (both upper and lower). Data obtained form literature do not allow establishing frequency range of isolated gingival involvement in MMP patients; in our series it was found in 33% of cases.

Since MMP gingival involvement has a so elevated frequency, its recognition during regularly performed periodontal practices could help both to decrease undiagnosed or misdiagnosed cases, and also to institute proper management.

However, in diagnosing isolated gingival cases, some problems can be faced because, especially in erosive cases, histopathological features are often non-conclusive because of the alteration caused by superimposed gingivitis or periodontitis, as previously reported for gingival cases of oral lichen planus, another common cause of DG (11).

It has been commonly reported that MMP affects older people, with a mean age of onset in the early to mid-60s, and female subjects are touched 1.5-2 times more often than males. In our series, females have been affected even more (f : m = 3.04 : 1), and for the first time we also reported that female subjects have a statistical change to be more affected in the gingival tissue, especially in the maxilla. On the contrary, male are less involved, but had more possibilities to develop also extra oral features. This could be an interesting issue for clinicians who have to deal with this type of disease, considering female patients more predisposed to gingival weakness and male patients probably presenting with a more severe form.

Retrospective observational studies such as the present may have limitations; however, we try to add evidence regarding the specific gingival involvement in MMP patients, which has rarely been reported; this could be crucial for not only oral medicine specialists but also primary dental healthcare personnel and periodontists.

By the way, this is the largest group, ever reported, of predominantly oral MMP patients describing its gingival features. Future larger prospective studies could probably give more valuable information.

## References

[1] Scully C, Lo Muzio L (2008). Oral mucosal diseases: mucous membrane pemphigoid. Br J Oral Maxillofac Surg.

[2] Di Zenzo G, Carrozzo M, Chan LS (2014). Urban legend series: mucous membrane pemphigoid. Oral Dis.

[3] Chan LS, Ahmed AR, Anhalt GJ, Bernauer W, Cooper KD, Elder MJ (2002). The first international consensus on mucous membrane pemphigoid: definition, diagnostic criteria, pathogenic factors, medical treatment, and prognostic indicators. Arch Dermatol.

[4] Chan LS (2001). Mucous membrane pemphigoid. Clin Dermatol.

[5] Leao JC, Ingafou M, Khan A, Scully C, Porter S (2008). Desquamative gingivitis: retrospective analysis of disease associations of a large cohort. Oral Dis.

[6] Lo Russo L, Fedele S, Guiglia R, Ciavarella D, Lo Muzio L, Gallo P (2008). Diagnostic pathways and clinical significance of desquamative gingivitis. J Periodontol.

[7] Arduino PG, Farci V, D'Aiuto F, Carcieri P, Carbone M, Tanteri C (2011). Periodontal status in oral mucous membrane pemphigoid: initial results of a case-control study. Oral Dis.

[8] Schellinck AE, Rees TD, Plemons JM, Kessler HP, River-Hidalgo F, Solomon ES (2009). A Comparison of the periodontal status in patients with mucous membrane pemphigoid: a 5-year follow-up. J Periodontol.

[9] Lo Russo L, Guiglia R, Pizzo G, Fierro G, Ciavarella D, Lo Muzio L (2010). Effect of desquamative gingivitis on periodontal status: a pilot study. Oral Dis.

[10] Gallagher G, Shklar G (1987). Oral involvement in mucous membrane pemphigoid. Clin Dermatol.

[11] Mignogna MD, Lo Russo L, Fedele S (2005). Gingival involvement of oral lichen planus in a series of 700 patients. J Clin Periodontol.

